# First basin scale spatial–temporal characterization of underwater sound in the Mediterranean Sea

**DOI:** 10.1038/s41598-023-49567-3

**Published:** 2023-12-20

**Authors:** Marta Picciulin, Antonio Petrizzo, Fantina Madricardo, Andrea Barbanti, Mauro Bastianini, Ilaria Biagiotti, Sofia Bosi, Michele Centurelli, Antonio Codarin, Ilaria Costantini, Vlado Dadić, Raffaela Falkner, Thomas Folegot, Daphnie Galvez, Iole Leonori, Stefano Menegon, Hrvoje Mihanović, Stipe Muslim, Alice Pari, Sauro Pari, Grgur Pleslić, Marko Radulović, Nikolina Rako-Gospić, Davide Sabbatini, Jaroslaw Tegowski, Predrag Vukadin, Michol Ghezzo

**Affiliations:** 1 CNR-National Research Council, ISMAR - Institute of Marine Sciences in Venice, Castello 2737/F, 30122 Venice, Italy; 2 CNR-National Research Council, IRBIM -Institute of Marine Biological Resources and Biotechnologies, SS Ancona, Largo Fiera Della Pesca 1, 60125 Ancona, Italy; 3 ARPA FVG — Regional Environmental Protection Agency of Friuli-Venezia Giulia, Via Cairoli 14, 33057 Palmanova, Udine, Italy; 4 IOR - Institute of Oceanography and Fisheries, Šetalište I. Meštrovića 63, 21000 Split, Croatia; 5 Blue World Institute of Marine Research and Conservation, Kaštel 24, 51551 Veli Lošinj, Croatia; 6 Quiet Oceans, Bâtiment Cap Ocean, Technopôle Brest-Iroise, 525 Avenue Alexis de Rochon, 29280 Plouzané, France; 7 Fondazione Cetacea Onlus, Viale Torino 7A, 47838 Riccione, Italy; 8 Faculty of Oceanography and Geography, University of Gdańsk, Av. Marszałka Piłsudskiego 46, 81-378 Gdynia, Poland

**Keywords:** Environmental sciences, Ocean sciences

## Abstract

Anthropogenic underwater noise is an emergent pollutant. Despite several worldwide monitoring programs, only few data are available for the Mediterranean Sea, one of the global biodiversity hotspots. The results of the first continuous acoustic programme run at a transnational basin scale in the Mediterranean Sea are here presented. Recordings were done from March 2020 to June 2021, including the COVID-19 lockdown, at nine stations in the Northern Adriatic Sea. Spatial–temporal variations of the underwater sound are described, having one third octave band sound pressure levels (SPLs) from 10 Hz to 20 kHz as metrics. Higher and more variable SPLs, mainly related to vessel traffic, were found close to harbours, whereas Natura 2000 stations experienced lower SPLs. Lower values were recorded during the lockdown in five stations. Median yearly SPLs ranged between 64 and 95 as well as 70 and 100 dB re 1 µPa for 63 and 125 Hz bands, respectively. These values are comparable with those previously found in busy shallow EU basins but higher levels are expected during a *business-as-usual* period. This is a baseline assessment for a highly impacted and environmental valuable area, that needs to be managed in a new sustainable blue growth strategy.

## Introduction

**Figure 1:**
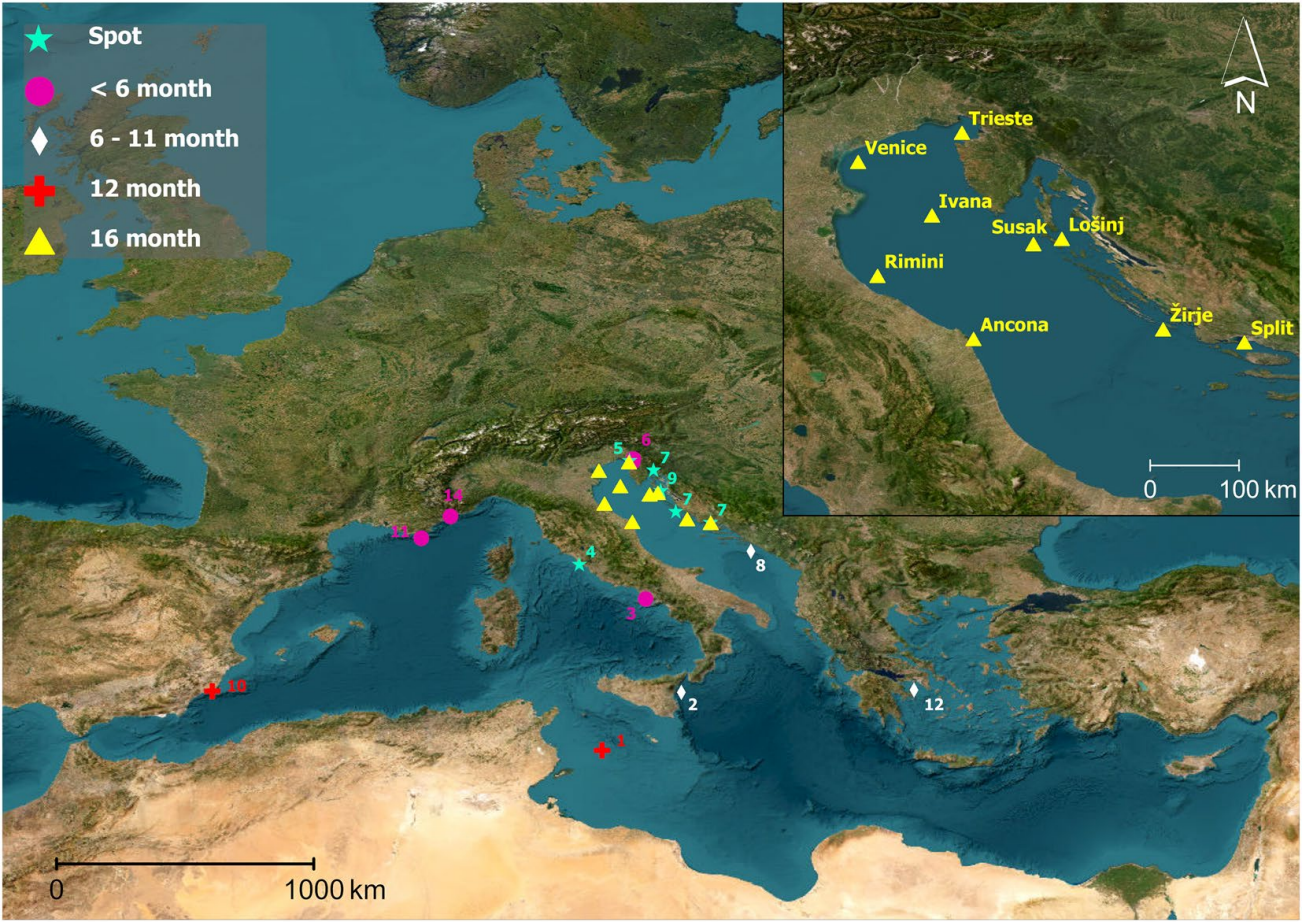
Geographical representation of published studies wherein underwater sound monitoring were described; different recording set-ups are highlighted: non continuous (spot) recordings are indicated with light blue stars and continuous monitorings are categorized on the base of the maximum duration of uninterrupted data collection (recording time coverage per each numbered station are given in Table S1 in Supplementary Materials). Data presented in this paper are acquired in the Northern Adriatic Sea stations, here represented with yellow triangles (SOUNDSCAPE project; see text for description), as also shown in the upper right panel of the figure.

Each underwater habitat is characterized by a unique soundscape, here defined as “the ambient sounds in terms of their spatial, temporal, and frequency attributes”^1^, that conveys important information related to environmental conditions, habitat quality and species presence^2^. Since the last century, however, human activities have significantly ensonified underwater soundscapes^3,4^ with negative impacts on marine life^5–8^. Nowadays underwater noise pollution has been recognized as a threat to marine ecosystems by international bodies^9^. As a result, many international projects have monitored underwater soundscapes in target areas worldwide, including Australia and U.S. waters^10–14^. From the first decade of the century several joint acoustic monitoring programmes have been developed in Europe as well^15^. This process has been facilitated by international agreements like the EU Marine Strategy Framework Directive (MSFD), the first legislation focusing on underwater noise pollution explicitly^15^. Despite this global effort, little information is still available about the underwater sound levels in the Mediterranean Sea, one of the global marine biodiversity hotspots^16^. The scientifically published data are geographically scattered, referring to a local scale and mostly related to a relatively short and/or non-continuous (spot) monitoring period (graphically reviewed in Fig. 1; for a more detailed description see Table S1, Supplementary Materials); in some cases, acoustic monitoring was run by or in compliance with national environmental agencies, e.g. the CALME program dedicated to the Western Mediterranean French coastline and the monitoring programme performed by the Regional Environmental Protection Agency of Friuli-Venezia Giulia in the Trieste Gulf (Italy).

Carrying out extended programmes of acoustic monitoring in the Mediterranean Sea is crucial: this is one of the busiest seas in the world, and both commercial and passenger traffic are expected to increase^17^; large portions of the basin appear to be chronically exposed to noise-producing human activities^18^ even though this semi-enclosed sea hosts a vulnerable marine biodiversity^16^. Here, species richness is most pronounced in western coastal and continental waters but also in the Aegean and Adriatic seas, with the latter showing high concentrations of endangered, threatened, or vulnerable species^16^. Specifically, the Northern Adriatic Sea (NAS) hosts numerous Natura 2000 sites as well as marine and coastal protected areas and it has been recognised as an Ecologically and Biologically Significant Area (Convention on Biological Diversity)^19^. At the same time, NAS is one of the underwater noise hot spots in the Mediterranean Sea^18^, experiencing intense marine traffic due to commercial shipping and fishing activities, known for its thriving tourism industry and for its exploitation by oil and gas companies, including hundreds offshore platforms^18,20^. NAS vulnerable biota is currently under the pressure of combined climatic and anthropogenic impacts^21,22^.

The Interreg project SOUNDSCAPE (Soundscapes in the North Adriatic Sea and their impact on marine biological resources) is the first continuous acoustic monitoring run at a transnational basin scale in the Mediterranean Sea. It aimed to document the NAS underwater soundscape through a shared institutional, technical and scientific collaboration of eight partners. This resulted into a coordinated monitoring network, that continuously collected underwater acoustic data from March 2020 to June 2021. Intra-basin comparison was assured by using the same type of automatic recorder and applying the same standard procedure in data collection and analysis. The monitoring stations (Fig. 1) were located close to the entrance of important harbours such as Venice (Italy; MS1), Trieste (Italy; MS4) and Split (Croatia; MS8). Other stations, however, were in proximity to important conservation areas like Natura 2000 sites (Monte Conero Regional Park, Italy; MS3; Losinj archipelago, Croatia, MS6) or fish reproductive areas (Žirje, Croatia; MS7). At the end, one station was placed outside the territorial waters, in the middle of NAS, to be close to the main shipping lanes going to Venice, Trieste and Koper ports, at an unmanned gas platform (Ivana, MS9). This platform collapsed during a heavy storm along the study period and the whole area was closed with restricted access; another dismissed platform was used as replacement location, just a couple of miles apart.

This study aims to describe the spatial and temporal variations of the ambient sound pressure levels recorded along the SOUNDSCAPE project over one year period, on the base of the recently released dataset^23^, and to discuss the results in the light of available literature. It is important to note that the presented results are referring to the peculiar 2020 year, which has been characterized by two epidemic COVID-19 waves (February and May 2020; October and December 2020) and an intermittent but year-long restriction of mobility.

## Results

**Figure 2:**
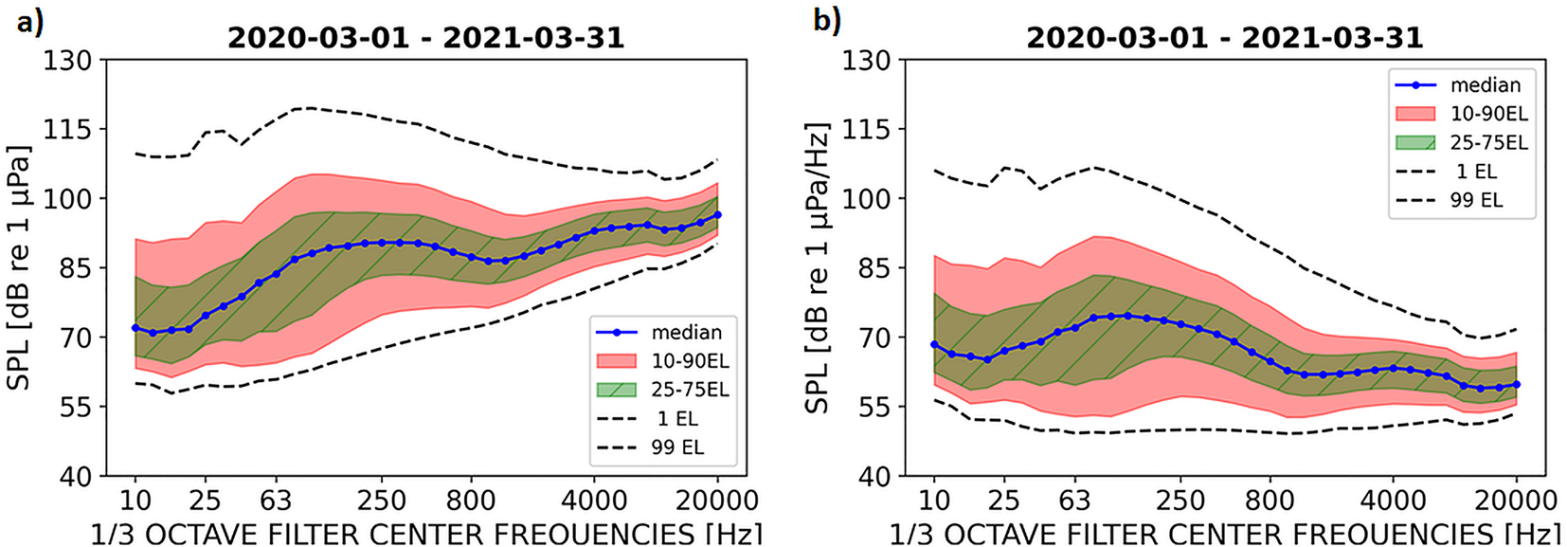
(**a**) One third octave band (base 10) SPLs calculated on the whole dataset, i.e. including all the acoustic files collected from 1 April 2020 to 31 March 2021 by each monitoring station: median values are shown in blue, SPLs included between the 10 and 90 Exceeded Levels (ELs) in pink, the Inter Quartile Range (25–75 ELs) in green and the 1 and 99 ELs band with dotted lines; (**b**) same spectra calculated by including the bandwidth correction.

The annual underwater sound pressure levels (SPLs) calculated on all the acoustic data collected in the nine NAS recording stations are concisely summarized in Fig. 2a, where the one third octave bands (base 10) (hereafter TOBs) spectra are shown, and in Fig. 2b, where SPL spectra are calculated by normalizing each TOB with a bandwidth correction. Overall, the median (50 Exceedance Level, EL; see Materials and Methods sections for detailed description) values range between 70 and 96 dB *re* 1 *µ*Pa, the TOBs centred from 250 to 400 Hz (90 dB *re* 1 *µ*Pa) and 6.3–8 kHz (94 dB *re* 1 *µ*Pa) as well as 16–22 kHz (96 dB *re* 1 *µ*Pa) showing the highest SPL values in the spectra (Fig. 2a). It must be noticed, however, that, when normalizing the metric, NAS recordings appeared to be mostly characterized by low frequencies contributions to the local soundscape (Fig. 2b).

Rare noisy events can be depicted from 10 EL, corresponding to the SPLs that are exceeded only 10% of the time. A concave curve represents the 10 EL and has a maximum value of 105 dB *re* 1 *µ*Pa at 125 Hz; even considering more rare noisy events (1 EL), SPL levels above 110 dB *re* 1 *µ*Pa are found only at frequencies lower than 2 kHz. A wide variation in the SPL range characterizes the low frequencies, having the lowest SPL values for 90 EL and, at the same time, the highest for 10 EL; in its turn this difference becomes smaller and gets centered towards the median value at higher frequencies.

### Underwater noise NAS inter-station spatial variability

**Figure 3:**
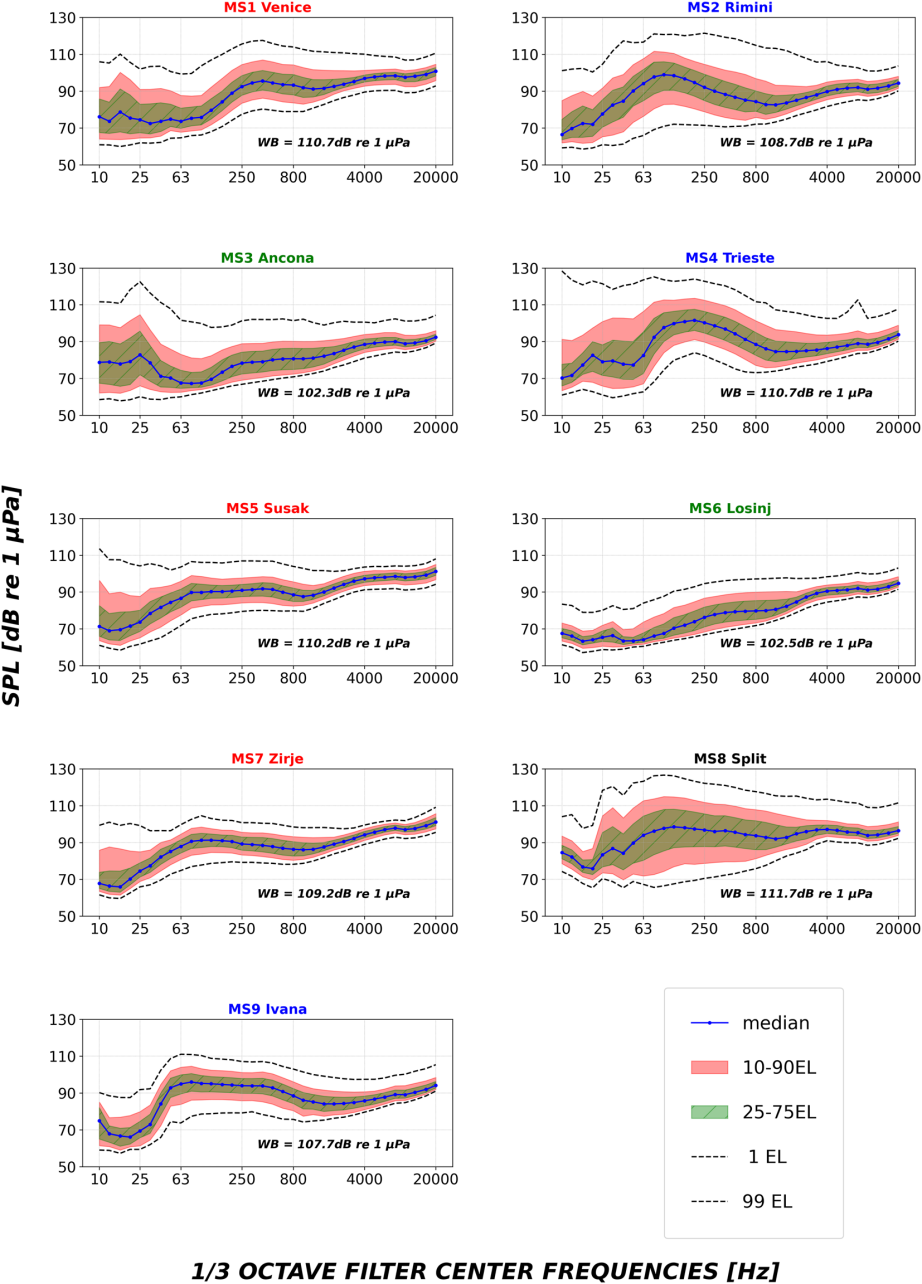
One third octave bands (base 10) SPLs (median, 10 and 90 Exceedance Levels) calculated per each of the nine NAS monitoring stations from 1 April 2020 to 31 March 2021; yearly median wideband SPLs (WB; 10 Hz–20 kHz) are also reported per station. Colours in the titles are related to the groups as identified in the text. Spectra calculated by including the bandwidth correction are provided in Fig. S1 (Supplementary Materials).

NAS monitored stations are characterized by different TOBs spectra contours (Fig. 3). Three different patterns can be generally recognized: (i) high median and 90 EL sound levels in the low frequency range (below 1 kHz) as the case of Rimini (MS2), Trieste (MS4), and Ivana (MS9), with high 10 EL SPLs being found in Trieste, only; (ii) a relatively flat contour in the low frequency range as the case of Susak-Losinj (MS5), Zrjie (MS7) and Venice (MS1); (iii) low median spectra SPLs in Ancona (MS3) and Losinj (MS6), which are the quietest stations of the network according to the yearly median wideband SPLs (10 Hz–20 kHz; Fig. 3). Split (MS8) shows the highest SPL variability among the stations, with reference to the low frequency TOBs.

### Underwater noise NAS intra-station temporal variability

**Figure 4:**
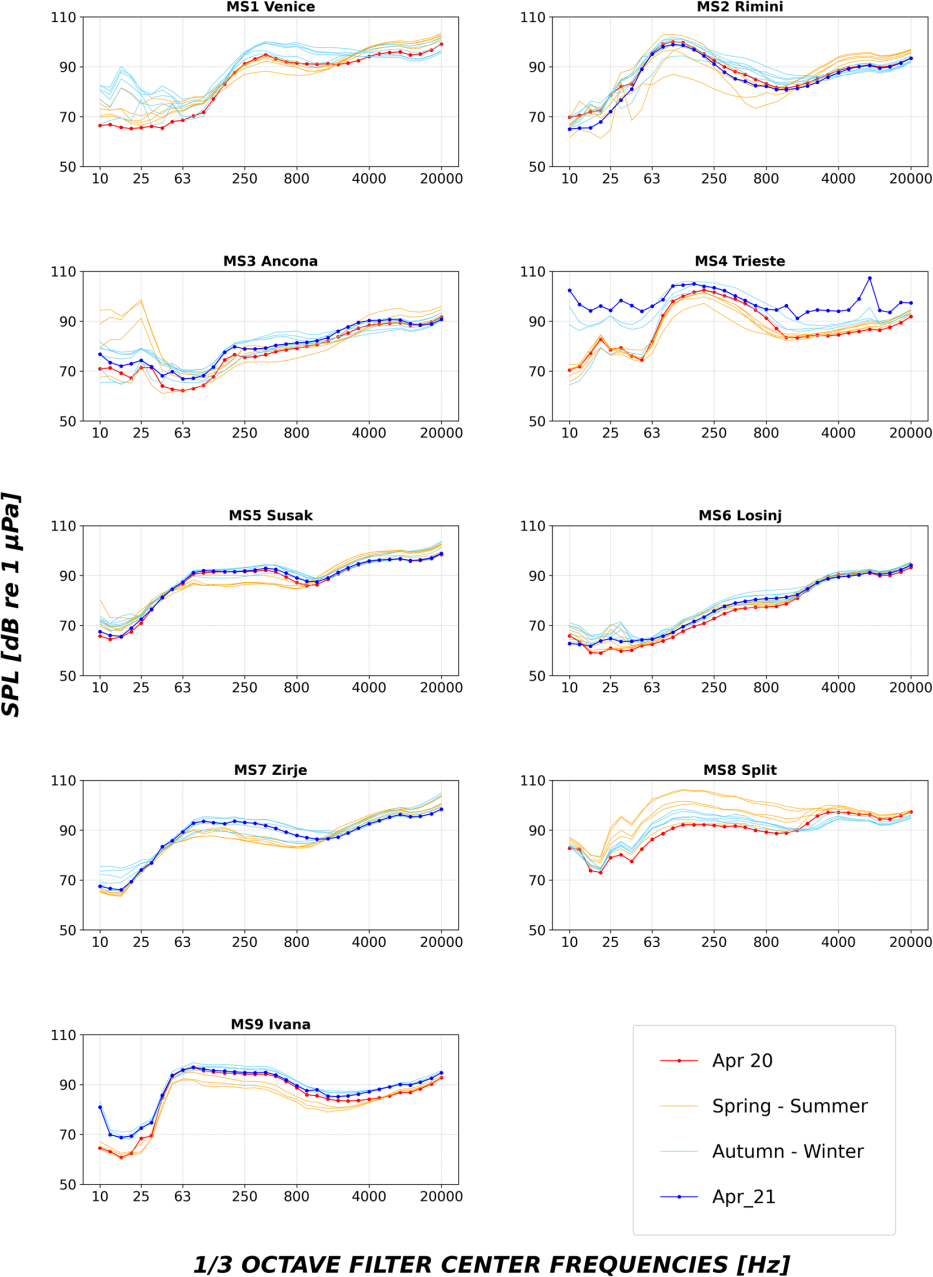
Monthly spectra (one third octave bands SPLs, median level) calculated per each NAS monitoring station. In orange are highlighted the spring–summer months (01/04/2020–30/09/2020) and in light blue the autumn–winter months (01/10/2020–31/03/2021). The month mostly characterized by the COVID19-Lockdown (April 2020) is indicated by the blue line; April 2021 (red line) is here added for comparisons. Spectra calculated by including the bandwidth correction are provided in Fig. S2 (Supplementary Materials).

For each monitoring station, monthly variations are shown in Fig. 4 as based on the median TOB spectra contours. In some cases (Venice, MS1; Trieste, MS4; Susak, MS5; Zrije, MS7) autumn–winter exceed spring–summer monthly median SPLs, mostly in the low frequency range. Interestingly, a seasonal difference between autumn–winter and spring–summer median SPL monthly values is generally higher at the 500 Hz TOB, known to be influenced by wind and weather conditions^24–26^, than at the 63 Hz TOB, here considered a proxy for ship-dominated underwater noise; this accords with a higher number of windy days (Beaufort scale higher than 5) recorded in autumn–winter compared to spring–summer in most of the stations, except Split (see Tables S2 and S3, Supplementary Materials). In fact, Split (MS8) has the opposite pattern (Fig. 4). Almost no temporal changes can be observed in Losinj (MS6). The seasonal spectra calculated as TOB SPLs (median level) per each NAS monitoring station are provided in the Supplementary Materials (Figs. S3 and S4, Supplementary Materials).

When considering temporal variations, it must be stressed that the period between middle March and middle May 2020 has been characterized by the COVID-19 pandemic outbreak and by the lockdown at national level both in Italy and Croatia (refer for example to a recent study^27^ for the consequences on maritime traffic). The lockdown restrictions induced a general reduction of the NAS vessel traffic, which is expected to be detectable in the recordings at the monthly timescale mostly in April 2020. According to Fig. 4, April 2020 median SPLs (highlighted in red) were lower than April 2021 median SPLs (highlighted in blue) in Trieste (MS4) and Ancona (MS3) on the Italian side and - partly - in Losinj (MS6) on the Croatian side. Although April 2021 recordings were not available in the case of Venice (MS1) and Split (MS8), April 2020 median SPLs were lower than the other months of the year in most of the considered frequencies.

### The MSFD shipping noise frequencies: 63 and 125 Hz one third octave band SPLs

The 63 Hz and 125 Hz TOBs were selected for a more detailed analysis, to compare them to other available studies: Table 1 summarized yearly median SPL and inter quartile range as well as the arithmetic mean in these bands for each monitoring station. Rimini (MS2), Split (MS8) and Ivana (MS9) show the highest mean and median SPL values, together with Trieste (MS4). Additionally, Rimini, Trieste and Split have the highest inter-quartile levels, indicating a high intra-site SPL variability.

**Table 1:** Median (50 EL, percentage exceedance level), Inter Quartile Range (IQR) SPLs (dB *re* 1 *µ*Pa) and arithmetic mean (AM) in the 63 Hz and 125 Hz one third octave bands for each monitoring stations calculated for one-year period (01/04/2020–31/03/2021).

	63 Hz			125 Hz		
	AM	50EL	IQR	AM	50EL	IQR
MS1 Venice (IT)	97	73	11	96	81	11
MS2 Rimini (IT)	105	94	17	109	98	15
MS3 Ancona (IT)	95	68	10	88	70	10
MS4 Trieste (IT)	111	83	23	111	100	13
MS5 Susak Losinj (HR)	97	87	10	96	90	7
MS6 Losinj (HR)	99	64	5	93	70	10
MS7 Zirje (HR)	92	88	7	95	91	7
MS8 Split (HR)	112	94	24	114	99	21
MS9 Ivana (HR)	100	95	10	99	95	8

For both frequency bands, a difference of about 30–33 dB was found between the medians of data collected at the quietest and the noisiest monitoring stations (MS6 Losinj *vs*. MS9 Ivana and MS6 Losinj *vs.* MS4 Trieste for 63 Hz and 125 Hz TOBs, respectively), confirming a high spatial variability of the low frequency underwater sound levels.

A difference between the arithmetic mean and median SPL values is visible for all the stations (Table 1); this difference is one indicator of the skewness of the distribution of sound pressure levels compared to medians^28^, with the arithmetic mean being more affected by outliers in the noise level distribution^29^, such as the brief and high amplitude human-generated events.

**Figure 5:**
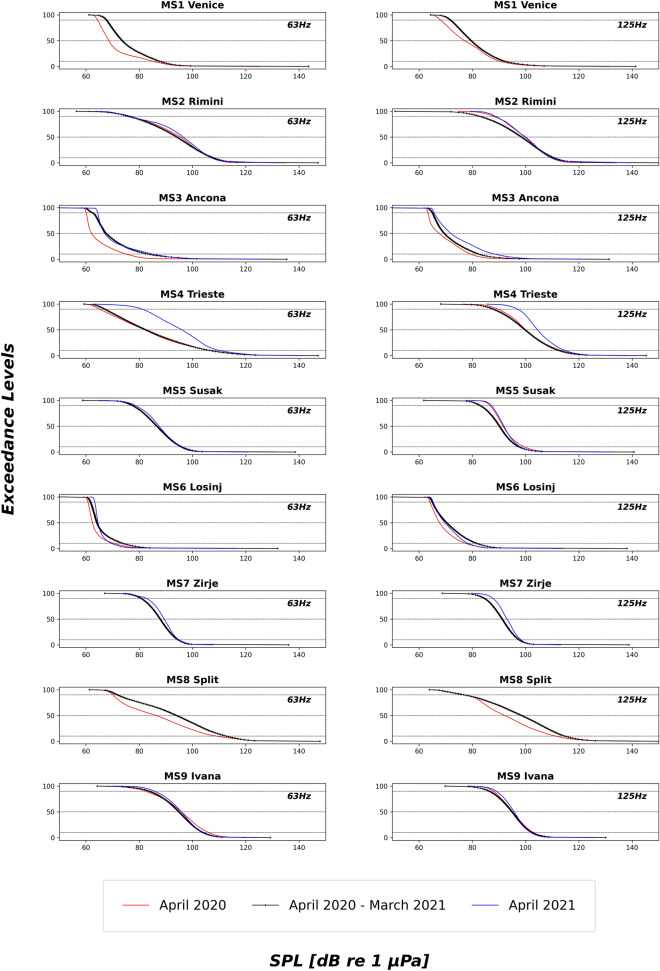
Underwater sound levels in NAS monitoring stations:SPLs (x-axis) values along all the Exceedance Levels (0-100th; y-axis) calculated for April 2020, April 2021 and for the whole year (1 April 2020 to 31 March 2021) (63 Hz one third octave band, left panel; 125 Hz one third octave band, right panel).

Figure 5 shows the distribution of SPLs for 63 and 125 Hz TOBs in April 2020 compared to those calculated over the whole monitoring year (April 2020 to March 2021) and over April 2021 for each station: Venice (MS1) and Split (MS8), on the Italian and Croatian NAS coastline, respectively, have the most pronounced reduction of SPLs in the 63 Hz and 125 Hz TOBs, together with Ancona (MS3) and Losinj (MS6) (see Table S4 in Supplementary Materials for comparisons between April 2020 *vs.* yearly median SPL values).

### AIS-Vessel marine traffic estimation in the study area

**Figure 6:**
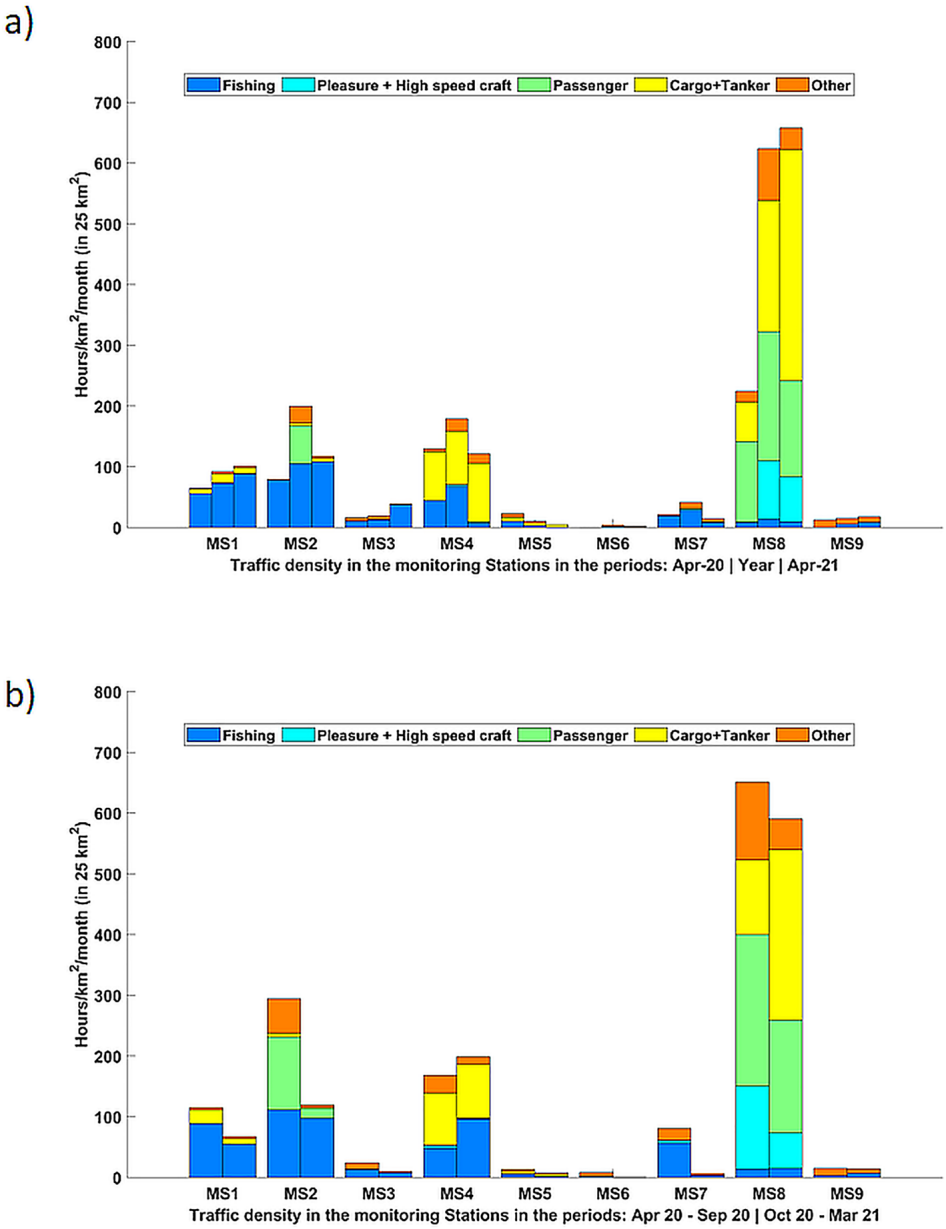
Average vessel density (here calculated in a 25 km square with the hydrophone position as a centre) per monitoring station for (**a**) a calendar year period (01/04/2020–31/03/2021), April 2020 (here considered as the most affected by COVID-19 induced lockdown) and April 2021 for comparison; (**b**) for the spring–summer (01/04/2020–30/09/2020) and autumn–winter (01/10/2020–31/03/2021) periods. MS1 Venice (IT); MS2 Rimini (IT); MS3 Ancona (IT); MS4 Trieste (IT); MS5 Susak Lošinj (HR); MS6 Lošinj (HR); MS7 Žirje (HR); MS8 Split (HR); MS9 Ivana D (HR).

During the here considered yearly period, the average vessel density was not equally distributed around the NAS considered stations, with Split (MS8), Rimini (MS2), Trieste (MS4) and Venice (MS1) being exposed to the most intensive shipping activity (Fig. 6a). A strong reduction in the vessel traffic is particularly visible in April 2020 (COVID-19 full lockdown period) compared to the average yearly vessel density and to the case of April 2021, with special reference to Split (MS8) and —partially – Venice (MS1) and Rimini (MS2) (Fig. 6a).

Seasonal differences in the vessel traffic can be highlighted for most of the stations (Fig. 6b): a clear increase of passenger and pleasure boats with AIS during the spring–summer is found in Rimini and Split together with an increase of the presence of the fishing vessels in Zrije (MS7). The number of fishing boats seems to remain constant in the other stations. Interestingly, a general reduction of vessel density was found along the autumn–winter season in some stations, as Venice (MS1), Rimini (MS2) and Split (MS8); this could be partially affected by different type of mobility restrictions that the Italian government introduced to address the second wave of COVID-19^30^. Overall, the whole monitoring year could be considered a peculiar year, not fully representative of *business-as-usual* NAS conditions.

## Discussion

The SOUNDSCAPE project represents the first spatially and temporally extensive research on underwater sound pressure levels run in the Mediterranean Sea, with a regional focus to the NAS. Median yearly SPL values calculated over the monitoring stations ranged between 70 and 96 dB *re* 1 *µ*Pa according to the considered frequency bands (in TOBs). A higher SPL variability was found in the low (approx. below 2–3 kHz) *vs.* higher frequencies (Fig. 2a) and lower frequencies contribution appears to be predominant in the monitored soundscapes (Fig. 2b). In its turn this suggests that noisiest events are mostly due to a mix of anthropogenic (boating and shipping) and abiotic (wind and waves) origins^25,26,31^.Still, multiple sources could be responsible for the observed pattern and further analysis will be dedicated to the characterization of biological and non-biological contributions to the local soundscapes.

High spatial variability in the underwater sound levels was found among the SOUNDSCAPE monitoring stations. This is highlighted by the sound spectra contours shown in the present research (Fig. 3): Split (MS8) showed the highest and more variable levels, together with Trieste (MS4), Rimini (MS2) and Venice (MS1). According to the presented elaboration of AIS available data (Fig. 6), Split is the most exposed station to intensive AIS-vessel traffic, together with Venice, Rimini and Trieste: a significant number of ships typically transports goods and oil and gas to industrial centres located in Split^32^ (Croatia), in Venice (Italy) and in the whole Trieste Gulf, with the ports of Trieste (Italy), Koper (Slovenia) and Rijeka (Croatia) as final terminals^33^. Apparently, Ivana (MS9), having high noise levels at low frequency, was less surrounded by close marine traffic compared to the other above-mentioned stations (Fig. 6); being located at open and deeper sea, however, Ivana is likely affected by distant noise sources. The stations of the network that experienced the lower sound pressure levels were Ancona (MS3) and Losinj (MS6); these stations were located close to or inside Natura 2000 sites and the acoustic data likely reflected a general low level of ship noise. Additionally, Losinj (MS6) was not impacted by ships' noise being physically separated from the open water shipping lanes due to its position inside the Cres-Losinj archipelago.

Observed variation in the spectra contour could also reflect the occurrence of abiotic events as weather conditions and wind: in shallow waters, distant sources are likely to be more attenuated and local sources, such as wind and waves, are more likely to dominate the soundscape^24^. NAS is characterized by winter outbreaks of cold air associated with the Bora events (North-Easterly wind), which stir up the entire water column over most of the area having a depth inferior to 50 m, i.e. practically most of the NAS basin. Dominant wind, in addition to Bora, is Scirocco (South-Easterly wind), which is predominant in the summer and early autumn^34^. Examining the wind events along the study period, a higher number of windy days (Beaufort scale higher than 5) were recorded in autumn–winter compared to spring–summer in most of the stations (Table S2, Supplementary Materials). Accordingly, the seasonal noise variation was more evident for the 500 Hz TOB compared to the 63 Hz and 125 Hz (Table S3, Supplementary Materials), confirming a general weather-driven rather human-generated seasonal change at the NAS soundscape. Higher sound levels below 2 kHz were depicted in the autumn–winter months in all the stations, except for Split (MS8; Fig. 4); this exception could be explained by Split being the most characterized by anthropogenic traffic, which, following our data, increased during the spring–summer period (Fig. 6b). The observed seasonal noise variation agrees with an earlier study^35^, that reports higher SPLs in the low frequency range during the winter compared to the summer periods, mainly related to the sea state*.*

Finally, the spatial–temporal variability of spectra contours could depend on the underwater noise propagation conditions, that are influenced by the water column properties, by bathymetry and seabed substrates in the study area. NAS oceanographic conditions are subject to strong seasonal variations: in late autumn and winter the shallow water column becomes vertically homogeneous because of direct wind mixing whereas thermal stratification gradually increases from spring to a maximum in August because of heat accumulation in the upper layers^36^. This implies a seasonal variation in the sound speed profile of the water column, which could, together with differences in sounds sources, explain seasonal variability in NAS underwater noise levels.

The here considered monitoring year included the COVID-19 pandemic event, which caused a disruption in shipping activities. Lockdown in Europe (including Italy and Croatia) was enforced mostly from the middle of March 2020 up until mid-May of the same year^27,37,38^. This mostly affected the maritime tourism industry as many European terminals reduced or stopped their operations and led to voluntary fishing cessation or reduction^27^, with a decrease of about 50% of fishing effort and a completely different spatial distribution of the fishing grounds^38,39^. Additionally, a general reduction of the density of dry and wet bulk carriers was reported in the Adriatic Sea and a 6% reduction in the ships speed was found in March–April 2020 compared to 2019^40^. A recently published paper^41^ further showed a temporary increase of the underwater noise emissions from the Baltic Sea and the Mediterranean Sea shipping in autumn 2020, after the June–July 2020 minimum; the acoustic emission however decreased again towards the end of 2020. This confirms the peculiar nature of the whole SOUNDSCAPE monitoring period.

Here a first, preliminary, exploration was dedicated to the SOUNDSCAPE data collected in April 2020, during the most strictly enforced COVID-19 lockdown period. Accordingly, a vessel traffic density reduction was found in April 2020 for all the SOUNDSCAPE monitoring stations compared to the other months of the year¸ with special reference to Split and—partially—Rimini (MS2; Fig. 6a). Variations in the TOBs levels in the April 2020 recordings were found for some but not all the monitoring stations, i.e. Venice (MS1), Ancona (MS3), Trieste (MS4), Losinj (MS6) and Split (MS8). If such a reduction of noise levels was expected for the case of harbours as Split and Trieste, it was more surprising to notice lower SPLs in Losinj and Ancona, which are usually characterized by a low AIS-vessel traffic. These two latter areas, however, typically host local and touristic recreational boating^42^. Small boat traffic was likely strongly reduced during COVID-19 related lockdown, although no data can be provided due to a lack of information on the distribution of small boat without AIS. Recreational noise often dominates coastal soundscapes^43,44^ and its possible reduction could eventually explain the observed results. The role of recreational boats as noise source for the NAS soundscape needs to be further investigated in future studies.

Finally, a dedicated focus on the yearly sound levels in TOBs with centre frequencies at 63 Hz and 125 Hz has been set to compare the SOUNDSCAPE recordings with other studies, being these frequency values the most available data in the literature. This is because they have been chosen as the indicator for low-frequency shipping continuous sound according to the EU's Marine Strategy Framework Directive (Descriptor 11.2)^45^. Overall NAS median SPLs ranged between 64 and 95 dB *re* 1 *µ*Pa and between 70 and 100 dB *re* 1 *µ*Pa at 63 and 125 Hz TOBs, respectively (Table 1). For comparative purposes, yearly median SPLs at 63 Hz and 125 Hz TOBs range between 65 and 115 dB *re* 1 *µ*Pa in the Baltic Sea^46^ and from 82 to 95 dB *re* 1 *µ*Pa in UK waters^29^. The SOUNDSCAPE dataset, however, has been recorded along a temporal period that included the COVID-19 lockdown period and in a year characterized by general restriction and precautionary reduction of traffic. This means that it is likely to find a noisier soundscape in a *business-as-usual* scenario. In general, SOUNDSCAPE values are lower than those obtained by the other long-term studies run in the Mediterranean Sea: for example, yearly median SPLs in the same TOB frequencies in the Marine Protected Area of Lampedusa (Italy) were found to be equal to about 100 dB *re* 1 *µ*Pa^35^. Even higher median SPLs calculated over 10 months characterized the Gulf of Catania^47^ (Italy) (112 and 107 dB *re* 1 *µ*Pa at 63 Hz and 125 Hz TOB, respectively); in this case, data were collected at the extreme water depth of 2100 m. Recently, hourly 63 and 125 Hz TOB median values have been found mostly between 90 and 105 dB *re* 1 *µ*Pa during the winter period (November-March 2019): here^48^, acoustic data were recorded at 1000 m depth in the South-eastern Adriatic Sea, to the South of Split (MS8), approximately at 20 km west of Dubrovnik (Croatia).

Monthly averaged SPLs ranged between 92 and 115 dB *re* 1 *μ*Pa at 63 and 125 Hz TOBs in the yearly measurements in the port of Cartagena^49^ (Spain) and median SPLs from 91 to 98 dB *re* 1 *µ*Pa at 63 Hz and 125 TOB s were recorded at the Gulf of Naples^50^ along three months period (Italy), both areas being under intensive urban settlements and maritime traffic. These sound levels are comparable to the SOUNDSCAPE levels recorded in port areas as Split (MS8) and the Trieste Gulf (MS4). Continuous short-time assessment run in the Trieste Gulf indicated also daily SPLs ranging between about 65 and 120 dB *re* 1 *µ*Pa at the 63 Hz and 125 Hz TOBs inside the Natural Marine Reserve of Miramare^51^ and average levels between 83 and 101 dB *re* 1 *µ*Pa at 63 Hz and 125 Hz TOBs in the Slovenian waters^52^.

Interestingly, the here reported SPLs in the 63 and 125 Hz TOBs are comparable to those obtained by shorter or non-continuous diurnal monitoring previous run in NAS suggesting that even shorter monitoring programmes could be informative where continuous long-lasting recordings are not available. Considering again, for example, the case of the Trieste Gulf (Italy), SOUNDSCAPE medians were lower than the yearly median SPLs calculated by a previous study^53^ for the 63 Hz TOB (83 *vs.* 94 dB *re* 1 *µ*Pa) but similar for the 125 Hz TOB (100 *vs*. 98 dB *re* 1 *µ*Pa). Similarly, average SPLs recorded continuously for about 4 days in the Port of Split lay at about 100–110 dB *re* 1 *µ*Pa at 63 and 125 Hz TOBs, respectively^32^ which is not too different from the yearly Split (MS8) SOUNDSCAPE median levels of 95 and 99 dB *re* 1 *µ*Pa, respectively. More variable median SPLs were found close to the Port of Civitavecchia, an important hub for maritime transport in the northern Tyrrhenian Sea (Italy)^54^, by a short-term diurnal underwater acoustic monitoring (from 63 to 145 dB *re* 1 *µ*Pa at 63 and 125 Hz TOBs).

Summing up, the present paper establishes baseline levels for the assessment of underwater noise future trends in the Northern Adriatic Sea, which is particularly relevant since marine traffic is expected to substantially grow in this area in the next decades. Here, we demonstrated that maritime traffic clearly affects the local coastal soundscape, leading to an increase in the sound levels: harbour areas as Split and Trieste showed higher noise levels among the investigated NAS stations whereas Natura 2000 sites appeared to experience the lowest acoustic pressure. By including the most restrictive COVID-19-induced lockdown phase (April 2020) and a monitoring year that is broadly characterized by a general reduction of mobility, the SOUNDSCAPE dataset provides a unique scenario of NAS soundscape where the anthropophony is mostly reduced and therefore it represents a benchmark for evaluating the consequences of anthropogenic activities. On the other hand, higher levels in the *business-as-usual* period are expected, claiming for further monitoring, and raising concern on new marine development as the case of windfarms already planned in the study area^55^. Finally, the SOUNDSCAPE project represents the first effort aiming to quantifying underwater noise pressure to include it into future cumulative assessment of pollution and to promote a knowledge-based management of the marine resources, as it can be the case of the Maritime Spatial Planning process tool^56^. It appears clear from the presented data, that such an approach should consider intra-basin spatial and temporal variations.

## Methods

### Data acquisition and data coverage

**Figure 7:**
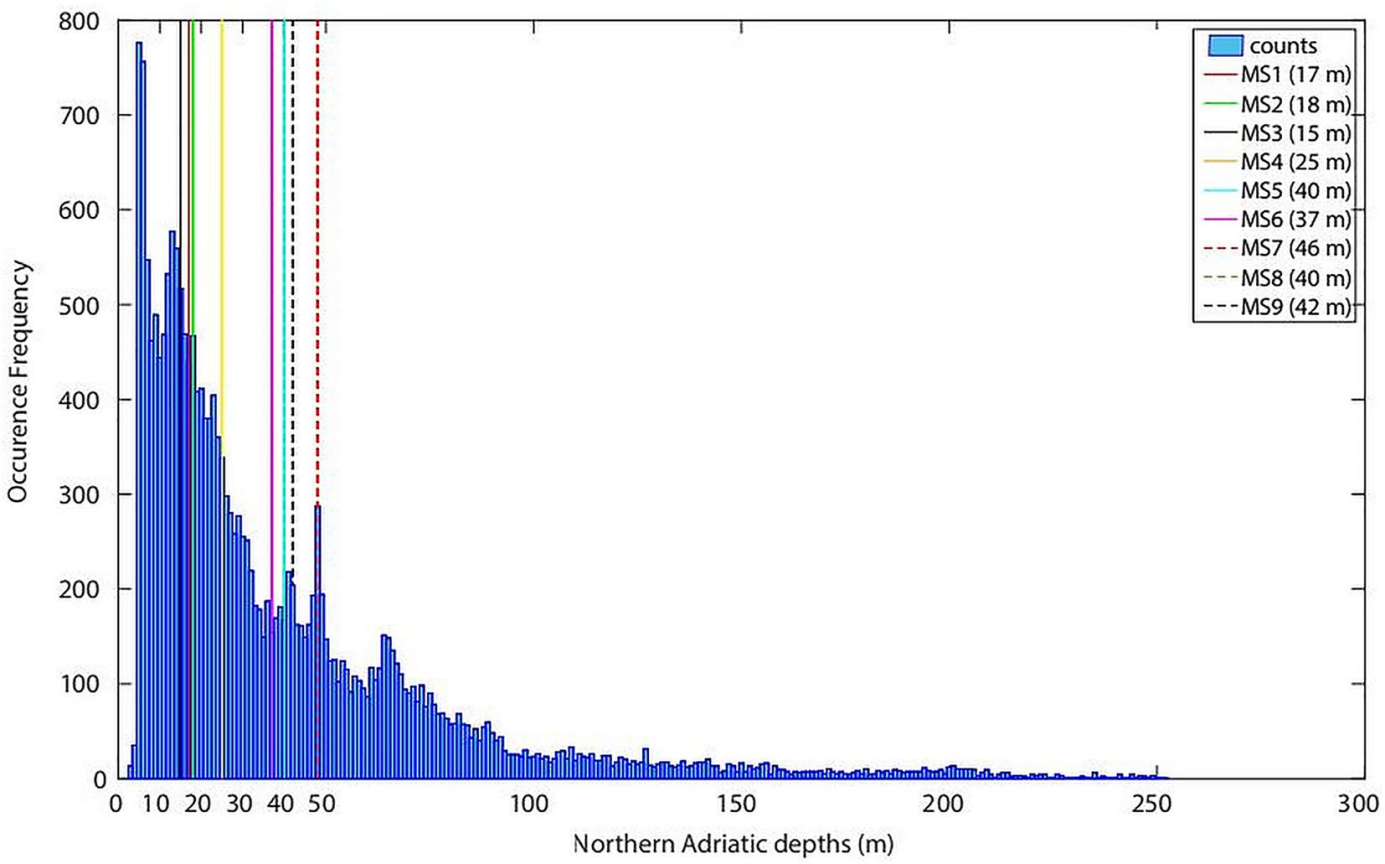
Distribution of the bathymetry in the study area (Northern Adriatic Sea). Bottom depth at each monitoring station of the SOUNDSCAPE project is highlighted.

Nine monitoring stations were set up (Fig. 1; Table S2, Supplementary Materials) encompassing different environmental characteristics, including water depth and bottom sediment types. The distribution of the water depth of the stations is representative of the shallow nature of the Northern Adriatic Sea (Fig. 7).

A consistent protocol was used by all the SOUNDSCAPE partners for data acquisition. Each monitoring station was equipped with the same instrument: an autonomous Develogic Sono.Vault stationary acoustic recorder, featured with a calibrated omnidirectional broadband Neptune Sonar D60 Hydrophone characterized by a sensitivity of − 192.7 dB *re* 1V/*µ*Pa (flat frequency response: 10 Hz–20kHz ± 3 dB), a programmable recorder, a battery set and a 1TB-SD memory card. The recorders were set to record continuously at a sampling rate of 44.1 kHz, providing a recording bandwidth of 22 kHz (16-bit resolution). The system calibration was checked in situ just before the deployment and after the recovery by using an air-pistonphone Grass 42AC (Grass Instruments, West Warwick, RI, USA), that generates a known sound pressure level at 250 Hz.

The same rig-design for mooring was applied throughout the study area: the recorders were anchored to the bottom about 3 m above the seabed and secured by polypropylene rope and extra flotations; an acoustic release was present in some stations. Attention was given to minimize the self-noise originated by the mooring and to proper locate the recorder deployments to assure no interaction with external infrastructures that could generate unwanted sounds.

Usually, a measurement period lasted 3 months, after which each recorder had to be recovered to download data and to remove biological fouling. The SOUNDSCAPE measurement period covers 16 months (from 1 March 2020 to 30 June 2021); the data coverage per each monitoring station is represented by Fig. S5 (Supplementary Materials) and it is described in detail by a previous study^23^.

### Acoustic and statistical analysis

The collected .wav files, for a total of 8.5 Tb of raw data, were stored on two servers at the research institutes of CNR Ismar (Venice, Italy) and IOR (Split, Croatia). No data compression was applied to the original files. The elaboration of all the collected data to obtain Sound Pressure Levels was performed by the same acoustic processing tool (Audio Noise Processing App; ANP) based on a Python code specifically developed for SOUNDSCAPE by the University of Gdansk together with CNR. Detailed information on the processing chain is given in a previously published research^23^. Used metric in this paper is the 20 s averaged SPLs data in the one third octave band (base 10), here defined according to the appropriate standards^57^.

A subset of data encompassing one year (01/04/2020–31/03/2021) was taken into consideration for the post-processing analysis. The selected time range includes continuous recordings representative of two set of oceanographic conditions, i.e. spring–summer (01/04/2020–30/09/2020) and autumn–winter (01/10/2020–31/03/2021). April 2021 data has been further separately analysed for comparisons with April 2020 recordings, a time characterized by the strictest COVID-19 lockdown.

The yearly data (grouped together and, subsequently, calculated per each single station) are initially represented as a TOBs spectra contours, where the exceedance levels (EL) are shown for each TOB. Exceedance levels provide an estimate of the distribution of sound levels over time^58^ and here have been calculated over the above-mentioned year. Thus, 10 EL (10% exceedance level) was the SPL value exceeded for the 10% of the year, usually representing rare sounds characterized by the highest noise levels. Accordingly, 90 EL can be assumed to describe noise levels during the local quietest condition, being a value that was almost always (90% of the year) exceeded along the year; 50 EL is the median level. Yearly median wideband (10 Hz–20 kHz) SPL values were additionally calculated per each station. Monthly variations have been further investigated by comparing the monthly median SPL levels.

Although all frequency bands were initially considered, a focus was also dedicated to the MSFD monitoring frequencies of 63 Hz and 125 Hz TOBs (Descriptor 11, Criterion 2^34^). Yearly (01/04/2020–31/03/2021) median and arithmetic mean (in accord to the recently released guidelines^59^) values were provided in agreement to TG Noise recommendations for monitoring underwater noise in European Seas^60^. Inter-quartile levels were also calculated as a metric to evaluate the SPL variability. Since the application of the most stringent measures against the spread of the COVID-19 pandemic concerns the SOUNDSCAPE recording period, the SPLs distributions measured in April 2020 for the 63 Hz and 125 Hz TOBs were also compared—for each monitoring station—to those measured in April 2021, the latter being more representative of unlocked usual conditions.

The TOBs spectra contours were also calculated by applying a correction factor (C_bw_T_) to normalize them with respect to each TOB bandwidth:

$${\text{C}}_{{{\text{bw}}\_{\text{T}}}} = \, - {1}0*{\text{log}}_{{{1}0}} \left( {{\text{Max F}}_{{\_{\text{T}}}} - {\text{Min F}}_{{\_{\text{T}}}} } \right),$$

where Max F__T_ and Min F __T_ are the first and the last frequency of each TOB T. Applying this correction, it is possible to assess for each TOB the mean contribution to the SPLs of the frequencies inside it.

### AIS-Vessel marine traffic estimation in the study area

An estimation of the NAS marine traffic was obtained from the Automatic Identification System (AIS) available data, that provides position and identification of ships of 300 gross tonnage and upwards, cargo ships of 500 gross tonnage and upwards and all passenger ships (whereas smaller recreational crafts can use AIS on voluntary base). Average ship traffic intensity within a 25 km^2^ area around each monitoring station, was extracted from EMODnet Human Activities web portal (www.emodnet-humanactivities.eu). EMODnet maps are based on AIS data yearly purchased from Collected Localisation Satellites (CLS) and ORBCOMM. The maps show shipping density in 1 × 1km cells of a grid covering all EU waters and some neighbouring areas. Density is expressed as hours per square kilometre per month and it is provided for different ship types. Data were available by month of the year and were downloaded for a period ranging from April 2020 to April 2021*.*

### Supplementary Information

Supplementary Information.

## Data Availability

The dataset of 20- and 60-s averaged Sound Pressure Levels (SPL) output files collected by SOUNDSCAPE and described in this paper is available on Zenodo (10.5281/zenodo.7472152). The Jupyter Notebook interactive document for data post-processing is freely available in ROHub, the Research object management platform (10.24424/hrhm-8849).
