# Author Correction: First basin scale spatial–temporal characterization of underwater sound in the Mediterranean Sea

**DOI:** 10.1038/s41598-024-52266-2

**Published:** 2024-01-19

**Authors:** Marta Picciulin, Antonio Petrizzo, Fantina Madricardo, Andrea Barbanti, Mauro Bastianini, Ilaria Biagiotti, Sofia Bosi, Michele Centurelli, Antonio Codarin, Ilaria Costantini, Vlado Dadić, Raffaela Falkner, Thomas Folegot, Daphnie Galvez, Iole Leonori, Stefano Menegon, Hrvoje Mihanović, Stipe Muslim, Alice Pari, Sauro Pari, Grgur Pleslić, Marko Radulović, Nikolina Rako-Gospić, Davide Sabbatini, Jaroslaw Tegowski, Predrag Vukadin, Michol Ghezzo

**Affiliations:** 1 CNR-National Research Council, ISMAR - Institute of Marine Sciences in Venice, Castello 2737/F, 30122 Venice, Italy; 2 CNR-National Research Council, IRBIM -Institute of Marine Biological Resources and Biotechnologies, SS Ancona, Largo Fiera Della Pesca 1, 60125 Ancona, Italy; 3 ARPA FVG — Regional Environmental Protection Agency of Friuli-Venezia Giulia, Via Cairoli 14, 33057 Palmanova, Udine, Italy; 4 IOR - Institute of Oceanography and Fisheries, Šetalište I. Meštrovića 63, 21000 Split, Croatia; 5 Blue World Institute of Marine Research and Conservation, Kaštel 24, 51551 Veli Lošinj, Croatia; 6 Quiet Oceans, Bâtiment Cap Ocean, Technopôle Brest-Iroise, 525 Avenue Alexis de Rochon, 29280 Plouzané, France; 7 Fondazione Cetacea Onlus, Viale Torino 7A, 47838 Riccione, Italy; 8 Faculty of Oceanography and Geography, University of Gdańsk, Av. Marszałka Piłsudskiego 46, 81-378 Gdynia, Poland

Correction to: *Scientific Reports* 10.1038/s41598-023-49567-3, published online 20 December 2023

The original version of this Article contained errors in the names of the authors Marta Picciulin, Antonio Petrizzo, Fantina Madricardo, Andrea Barbanti, Mauro Bastianini, Ilaria Biagiotti, Sofia Bosi, Michele Centurelli, Antonio Codarin, Ilaria Costantini, Vlado Dadić, Raffaela Falkner, Thomas Folegot, Daphnie Galvez, Iole Leonori, Stefano Menegon, Hrvoje Mihanović, Stipe Muslim, Alice Pari, Sauro Pari, Grgur Pleslić, Marko Radulović, Nikolina Rako-Gospić, Davide Sabbatini, Jaroslaw Tegowski, Predrag Vukadin and Michol Ghezzo, which were incorrectly reversed.

The original Article has been corrected.

