# Supplementary material for: First basin scale spatial–temporal characterization of underwater sound in the Mediterranean Sea
Source: Sci Rep. 2023 Dec 20;13:22799. doi: 10.1038/s41598-023-49567-3 (PMC10739739; doi:10.1038/s41598-023-49567-3)

Supplementary materials

Picciulin et al.

First yearly assessment of underwater sound levels at a basin scale in the Mediterranean Sea

Science of Total Environment

Table S1. List of published studies on underwater sound monitoring carried in the Mediterranean Sea: time coverage and acoustic recording set-up are highlighted. ID number refers to recording stations as shown in Figure 1 of the manuscript.

ID	Country	Time Coverage	Recording Set-Up	Reference
1	Italy	7-1-2013 / 6-30-2014	continuous (2 minutes on/28 minutes off)	Buscaino et al. 2016
2	Italy	7-2-2012 / 5-10-2013	continuous	Viola et al. 2017
3	Italy	9-2017 / 11-2017	continuous (1 minutes on/4 minutes off)	Pieretti et al. 2020
4	Italy	5-2017 / 9-2017	spot (60-s per station; monthly monitoring)	Cafaro et al. 2018
5	Italy	1-2012 / 12-2012	spot (15-min per station; monthly monitoring)	Codarin and Picciulin 2015
6	Italy	11-20-2020 / 2-13-2021	continuous	Diviacco et al. 2021
7	Croatia	4-2013 / 8-2013	spot (5 to 30 minutes per station)	Vukadin 2016
8	Croatia	10-2018 / 5-2019	continuous	Širović and Holcer 2020
9	Croatia	2007 / 2009	spot (5 minutes - ad libitum per session)	Rako et al. 2013
10	Spain	1-2014 / 12-2014	continuous	Rodrigo-Saura et al. 2019
11	France	5-2015 / 12-2018	continuous (5 minutes on/20 minutes off)	Poupard et al. 2022
12	Greece	02-2013 / 10-2013	continuous	Prospathopoulos et al. 2017
13	Slovenia	2-2015	continuous	Popit 2021
14	France	5-23-2019 / 8-1-2019	continuous	Di Franco et al. 2023

Table S2. Coordinates, bottom depth, sediment types of the recording locations and number of daily events having wind speed over the threshold of 10 m/s. Wind speeds were obtained by extrapolation from the SHYFEM modelling applied to the monitoring stations in the considered target period.
** Ivana D gas production platform collapsed in December 2020 , becoming no longer accessible; the two MS9 positions are few miles apart and the differences in the data collected are regarded not relevant.*

Monitoring station	Position		Depth (m)	Sediment type	N. of days with daily average wind speed > 10 m/s Autumn-Winter	N. of days with daily average wind speed > 10 m/s Spring-Summer
	Longitude (E)	Latitude (N)				
MS1 Venice (IT)	12°30.883’	45° 19.383’	17	Sand	13	4
MS2 Rimini (IT)	12°42.656’	44°10.254’	18	sand	7	2
MS3 Ancona (IT)	13°40.932’	43°31.954’	15	sand	4	0
MS4 Trieste (IT)	13°33.917’	45°37.095’	25	sandy mud	29	9
MS5 Susak Lošinj (HR)	14°17.293’	44°29.545’	40	rocks/sand	14	6
MS6 Lošinj (HR)	14°34.51’	44°32.747’	37	sand	25	10
MS7 Žirje (HR)	15°36.020’	43°37.788’	46	gravelly sand	15	8
MS8 Split (HR)	16°25.336’	43°29.895’	40	slightly sandy mud	14	8
MS9 Ivana D (HR)	13°15.720'	44°46.953’	42	terrigenous sand	14	11
MS9 Ivana E (HR)	13°14.674'	44°46.684’	42	terrigenous sand	-	-

Table S3. Difference (dB re 1 µPa) between autumn-winter and spring-summer median SPL values at the (i) 63 and (ii) 500 Hz 1/3 octave bands (TOBs); MS1 Venice (IT); MS2 Rimini (IT); MS3 Ancona (IT); MS4 Trieste (IT); MS5 Susak Lošinj (HR); MS6 Lošinj (HR); MS7 Žirje (HR); MS8 Split (HR); MS9 Ivana D (HR).

	**MS1**	**MS2**	**MS3**	**MS4**	**MS5**	**MS6**	**MS7**	**MS8**	**MS9**
63 Hz TOB<br>ship-noise dominated	-0.4	3.3	1.6	10.7	1.8	1.1	0.8	-4.5	1.9
500 Hz TOB<br>Weather dependent	5.3	7.3	3.8	6.5	5.9	2.1	6.5	-2.5	5.7

Table S4. Difference (dB re 1 µPa) between the yearly median (April 2020 - March 2021) and the April 2020 median SPLs in the 63 Hz and 125 Hz 1/3 octave bands

Monitoring stations	Median SPL variation (dB re 1 µPa)	
	63 Hz	125 Hz
MS1 Venice (IT)	5.1	2.5
MS2 Rimini (IT)	-1.3	-1.2
MS3 Ancona (IT)	5.5	1.9
MS4 Trieste (IT)	0.3	-0.3
MS5 Susak Lošinj (HR)	-0.2	-1.3
MS6 Lošinj (HR)	1.7	2.7
MS7 Žirje (HR)	n.a.	n.a.
MS8 Split (HR)	7.7	6.3
MS9 Ivana D/E (HR)	-1	-0.2

Figure S1. *One third octave bands (base 10) SPLs (median, 10 and 90 Exceedance Levels) calculated per each of the nine NAS monitoring stations from 1 April 2020 to 31 March 2021. Colours in the titles are related to the groups as identified in the text. Spectra are calculated by including the bandwidth correction.*

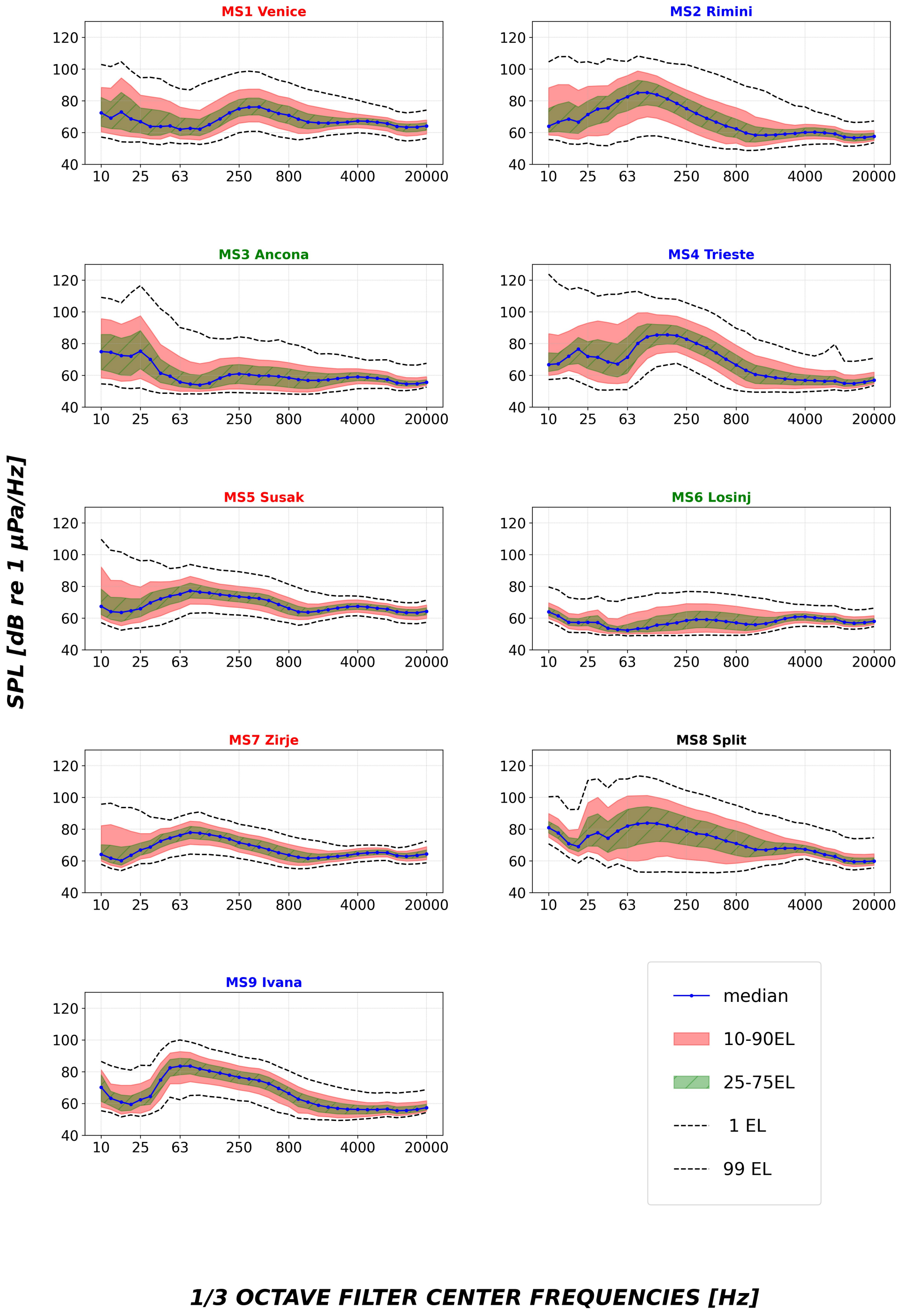

Figure S2. Monthly spectra (one third octave bands SPLs, median level) calculated per each NAS monitoring station. In orange are highlighted the spring-summer months (01/04/2020 - 30/09/2020; red line) and in light blue the autumn-winter months (01/10/2020 -31/03/2021; blue line). The month mostly characterized by the COVID19 related lockdown (April 2020) is indicated in blue. April 2021 (in red) is here added for comparisons. Spectra are calculated by including the bandwidth correction.

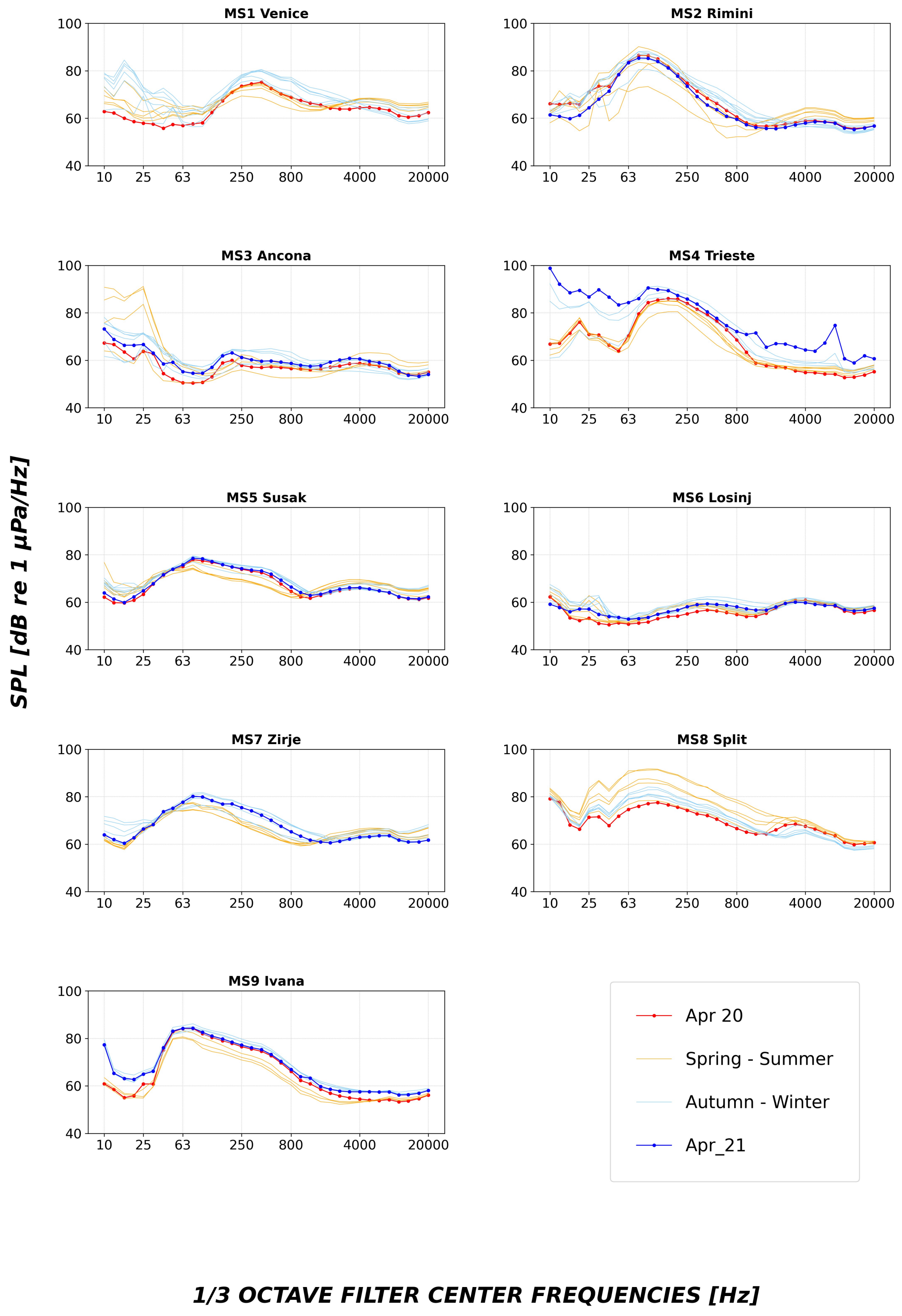

Figure S3. Seasonal spectra (one third octave bands SPLs, median level) calculated per each NAS monitoring station; spring-summer (01/04/2020 - 30/09/2020; red line) and autumn-winter months (01/10/2020 -31/03/2021; blue line).

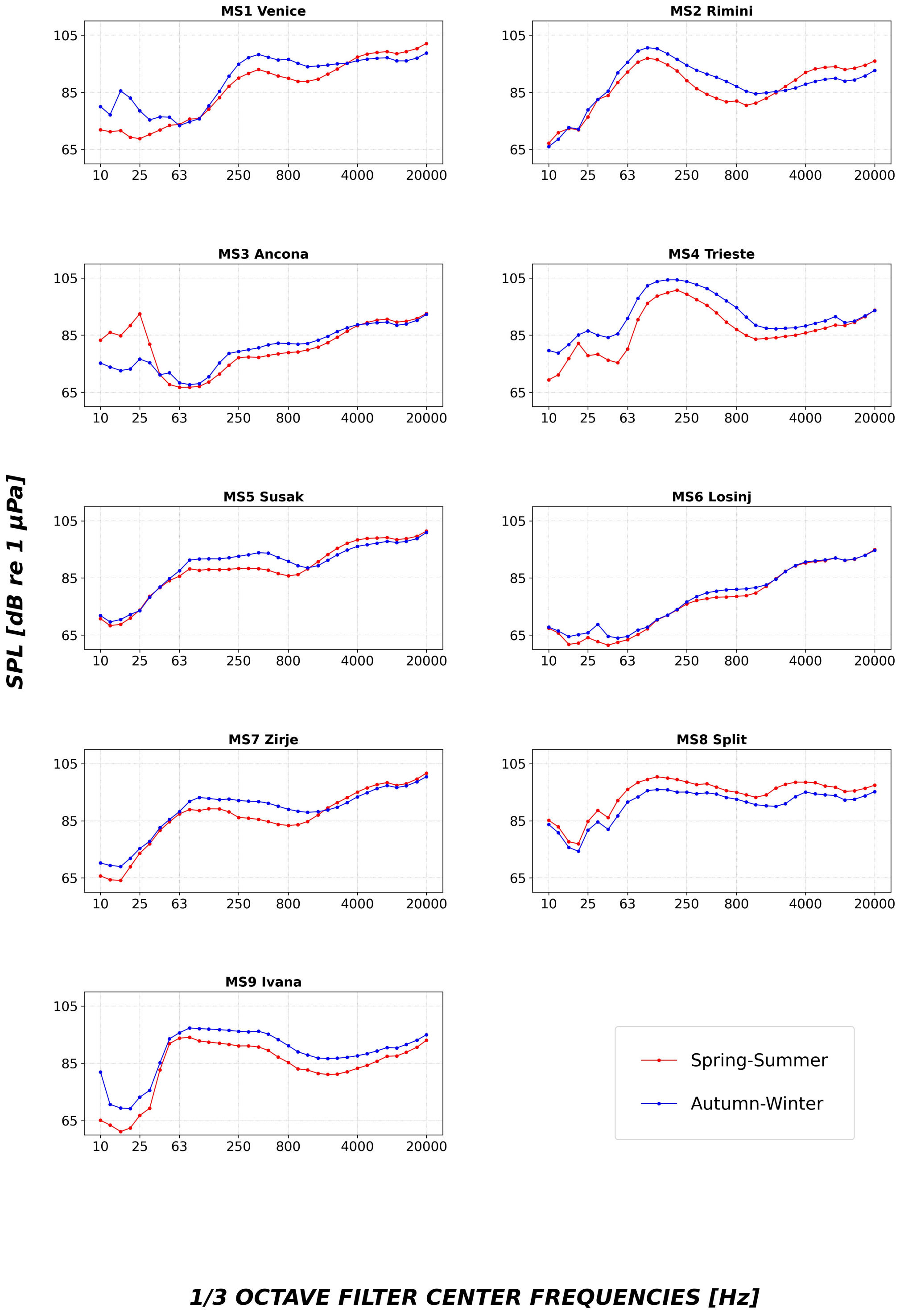

Figure S4. Seasonal spectra (one third octave bands SPLs, median level) calculated by including the bandwidth correction per each NAS monitoring station; spring-summer (01/04/2020 - 30/09/2020; red line) and autumn-winter months (01/10/2020 -31/03/2021; blue line).

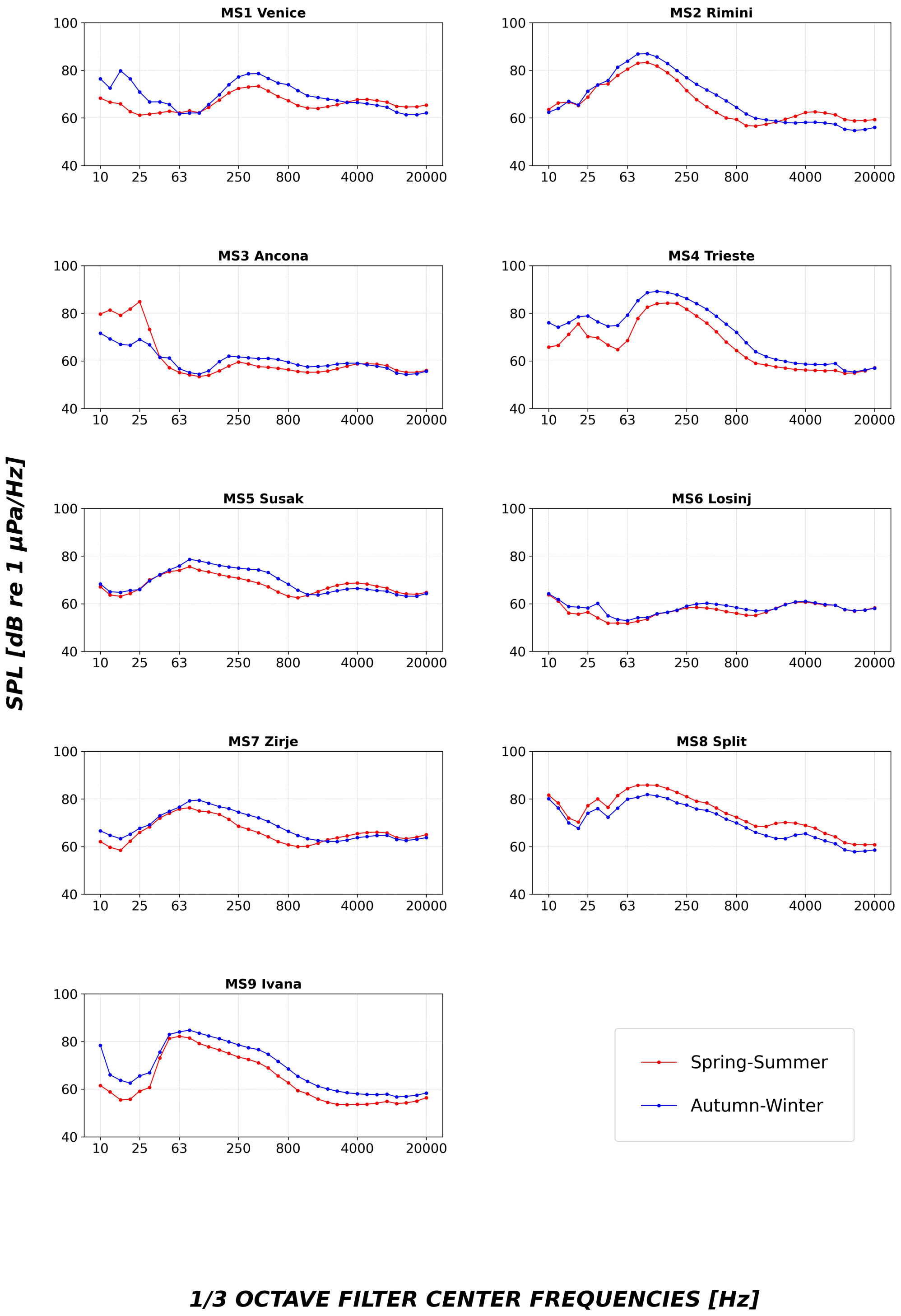

Figure S5. Data coverage per each recording station starting from end of February 2020 to the end of July 2021: data are available only for periods coloured in green, in red the data are missing¸ MS1 Venice (IT); MS2 Rimini (IT); MS3 Ancona (IT); MS4 Trieste (IT); MS5 Susak Lošinj (HR); MS6 Lošinj (HR); MS7 Žirje (HR); MS8 Split (HR); MS9 Ivana D (HR).

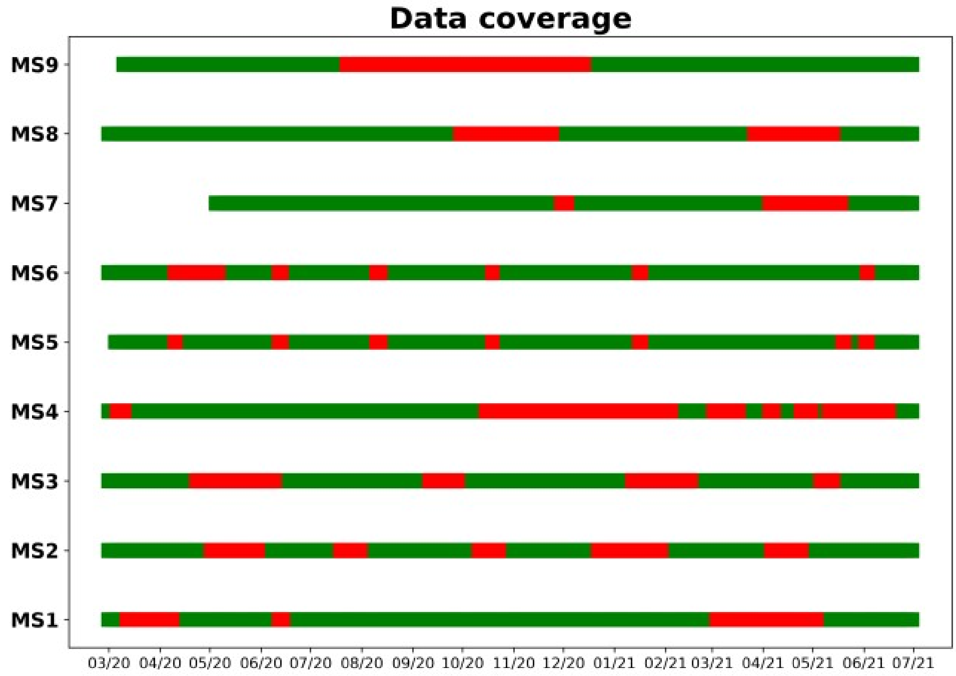

Supplement: Supplementary file 1 — Supplementary Information. [file 41598_2023_49567_MOESM1_ESM.pdf]
